# From Oxford to Hawaii Ecophysiological Barriers Limit Human Progression in Ten Sport Monuments

**DOI:** 10.1371/journal.pone.0003653

**Published:** 2008-11-05

**Authors:** François-Denis Desgorces, Geoffroy Berthelot, Nour El Helou, Valérie Thibault, Marion Guillaume, Muriel Tafflet, Olivier Hermine, Jean-François Toussaint

**Affiliations:** 1 IRMES, Institut de Recherche Médicale et d'Epidémiologie du Sport, INSEP, Paris, France; 2 Université Paris Descartes, Paris, France; 3 INSERM U909, université Paris Descartes, IFR 69 Paris Sud Université, Villejuif, France; 4 Service d'hématologie Hôpital Necker and CNRS UMR 8147, Paris, France; 5 CIMS, Hôtel-Dieu, Assistance Publique - Hôpitaux de Paris, Paris, France; Universidad Europea de Madrid, Spain

## Abstract

In order to understand the determinants and trends of human performance evolution, we analyzed ten outdoor events among the oldest and most popular in sports history. Best performances of the Oxford-Cambridge boat race (since 1836), the channel crossing in swimming (1875), the hour cycling record (1893), the Elfstedentocht speed skating race (1909), the cross country ski Vasaloppet (1922), the speed ski record (1930), the Streif down-hill in Kitzbühel (1947), the eastward and westward sailing transatlantic records (1960) and the triathlon Hawaii ironman (1978) all follow a similar evolutive pattern, best described through a piecewise exponential decaying model (*r* ^2^ = 0.95±0.07). The oldest events present highest progression curvature during their early phase. Performance asymptotic limits predicted from the model may be achieved in fourty years (2049±32 y). Prolonged progression may be anticipated in disciplines which further rely on technology such as sailing and cycling. Human progression in outdoor sports tends to asymptotic limits depending on physiological and environmental parameters and may temporarily benefit from further technological progresses.

## Introduction

World records (WR) highlight the progression of human performance. Our group recently analyzed WR from 5 measurable Olympic disciplines and demonstrated a major global fading in WR progression following a piecewise exponential decaying pattern [1]. Track and field, weight lifting or swimming competitions take place in a finite context with standardization of competitive fields, controlled environmental factors and major influence of physiological capacity on performance whereas outdoor sports are usually considered to be more influenced by environmental conditions, material or technical constraints. Therefore, outdoor events among the oldest and most popular have been ignored by previous studies that have analyzed human performance evolution [1], [2].

Performance depends on trainable variables (related to physiology, psychology, biomechanics, tactics) and other factors beyond the athlete's control (genetics, environment, climatic conditions) [3]. Environmental factors may modify results according to the discipline rules, mobility milieu (snow, ice, water, air), motion type (running, cycling, skiing, swimming) or duration of the event. As a result, International Federation of Rowing Associations, as well as other federations do not provide world records, though events “best times” are available [4]. We hypothesized that performance evolution of outdoor sports events would also follow a piecewise exponential decaying pattern.

## Materials and Methods

Ten outdoor events among the most popular in the world, performed in non motorized sports and presenting large variations in duration, longevity, competitive circumstances and frequency were analysed (**Table 1**). The Oxford-Cambridge rowing race is challenged since 1829, with an unchanged course against the streams of the Thames from “Putney to Mortlake” since 1845 [5], [6]. The Vasaloppet is a Swedish long distance cross-country skiing race held on the first Sunday of March between the village of Sälen and town of Mora for 90 km [6], [7]. Since 1909 the speed skating race “Elfstedentocht” takes place in the canals of the Dutch Friesland when ice freezes over the 200 km route around Leeuwarden [6], [8]. The record of transatlantic crew sailing eastward from New-York “Ambrose Light” tower to English “Lizard Point” (5417 km) was first set by Charlie Barr's crew in 1905 but the record has only been challenged for a few decades starting with Eric Tabarly [6], [9]. The oldest solo ocean race is challenged sailing transatlantic westward every four years from Plymouth to New England (Newport or Boston, orthodromic track: 5185 km) starting with Sir Francis Chichester in 1960 [6], [9]. The speed ski record is challenged since 1930 in varied places (Portillo, Chile; Silverton, USA; Les Arcs, France) and determined in a timing zone 100 meters long [6], [10]. The hour cycling record is accounted as firstly challenged outdoor in Paris since 1876 and registered by the International Cyclist Union (ICU) and the International Human Powered Vehicle Association (IHPVA) with different acceptance in the bicycle type used for the record [6], [11], [12]. The Streif (Kitzbühel, Austria) down-hill ski is 3312 m long with an 27% average gradient, held since 1930 and part of the ski world cup since 1967 [6], [13]. The Hawaiian Ironman is the first modern long-distance triathlon (3.86 km swimming, 180.2 km cycling and 42.2 km running). Starting in 1978, each year it is considered to be the World championship in long-distance triathlon [6], [14]. The channel crossing swim is usually challenged between Shakespeare beach (Dover, England) to “Cap gris nez” (France) over 33.8 km; firstly held in 1875 it has been regulated since 1927 [6], [15]. For all events, best performances (BP) only (equivalent to the race record) are accounted for into the analysis [5]–[15].

**Table 1:** Event parameters: longevity, occurrence and available performance number.

Event	Sport	Dates	Frequency	Performance number	
				total	best performance
**Oxford-Cambridge**	Rowing	1829	Annually	153	19
**Transatlantic record**	Crew sailing	1980	Free	11*	11
**Transatlantic record**	Solo sailing	1960	Every four years	13	9
**Channel crossing**	Swimming	1875	Free	772	16
**Ironman Hawaii**	Triathlon	1978	Annually	30	12
**Hour cycling record UCI**	Cycling	1876	Free	29*	29
**Hour cycling record IHPVA**	Cycling	1933	Free	20*	20
**Elfstedentocht**	Speed ice skating	1909	Annually	16	9
**Vasaloppet**	Cross-country ski	1922	Annually	86	17
**Speed ski record**	Down-hill ski	1930	Free	32*	32
**Streif down-hill**	Down-hill ski	1930	Annually	74	20

* performance is only registered when the best performance is improved.

### Function description and prediction

We performed BP modelisation as previously reported [1]: performance series for each event are fitted by the following function: *y_j_* (t) = *ΔBP* exp^(−aj.t′)^+*b; w*here *ΔBP* = *BP_i,j_*−*BP_f,j_* is an event indicator for the studied *j* period; *BP_i,j_* and *BP_f,j_* are the initial and final BP values respectively; *a_j_* is the positive curvature factor given by non linear regression; *b* is the asymptotic limit. Coefficients *a, b* and estimated predictions (years, asymptotic values) are obtained through an iterative algorithm.

The ratios *β* (progression range since the beginning of the event) and *β′* (present progression range) were calculated to describe the improvement over the final time frame in each event by using the following equation: *β = *(BP_i_/*b*)×100 and *β′ = *(BP_f_/*b*)×100.

For each event, this piecewise exponential decaying model provides successive periods. A period refers to a time slot defined by a group of consecutive BP, following a rupture of incline. A procedure based on the best adjusted *r^2^* was used to split BP series into periods. The algorithm was initiated by the first three BP values. The series was iteratively fitted by adding the next BP point using the presented above equation equation. For each fit, we obtained the adjusted *r^2^*; local maxima provide the changes of incline corresponding to the beginning of a new period. The minimum period duration is 6 years and the minimal BP number is three per period.

During the period *j*, parameters *a_j_* and *b_j_* were estimated using the Levenberg-Marquardt algorithm (LMA) [16] in a non linear least-squares regression to fit the model to BP. Coefficients of the prediction equation were calculated for the last period of each event through LMA with credibility interval [17]. The result was used to estimate the year *t* when the 99.95% (1/2000) limit is reached.

Data are expressed as mean±standard deviation.

## Results

The collected data provided a mean of 17.6±7.1 BP per event. Events had large differences in their initial progression range (*β* = 46.27±13.5%). Present mean achievement of the asymptotic performance, *β′*, was 94.6±7.%2 with seven events over 97%. Events with the lowest *β* and *β′* coefficients were: sailing transatlantic records, Streif and speed ski record and IHPVA hour cycling record (gathered in group 1: *β_1_* = 37.5±9.5%; *β′_1_* = 90.4±8.8%) whereas group 2 sport events: Oxford-Cambridge, Vasaloppet, Elfstedentocht, Ironman, Channel crossing and ICU Hour cycling record had higher values (*β_2_* = 53.5±12.1%; *β′_2_* = 98.0±3.2%).

**Figure 1:**
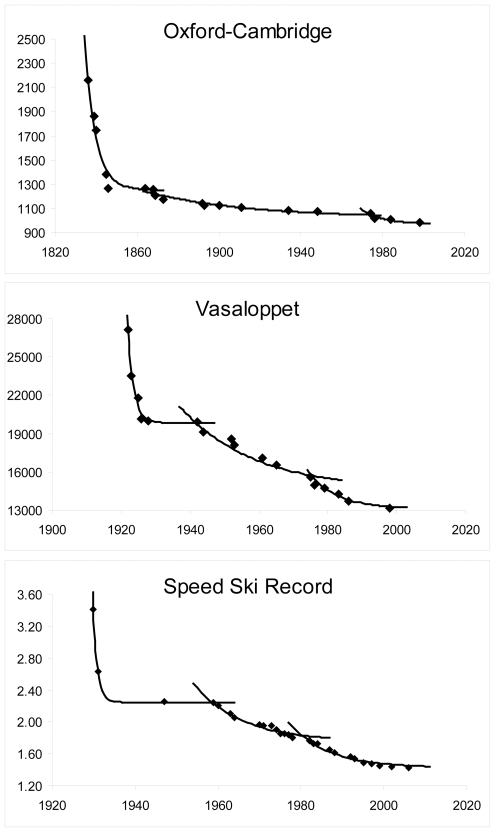
Model fitting for events with three progression periods. Performances of the Oxford-Cambridge boat race, Vasaloppet cross-country ski and speed ski record (for covering a 100-m distance) in seconds.

**Figure 2:**
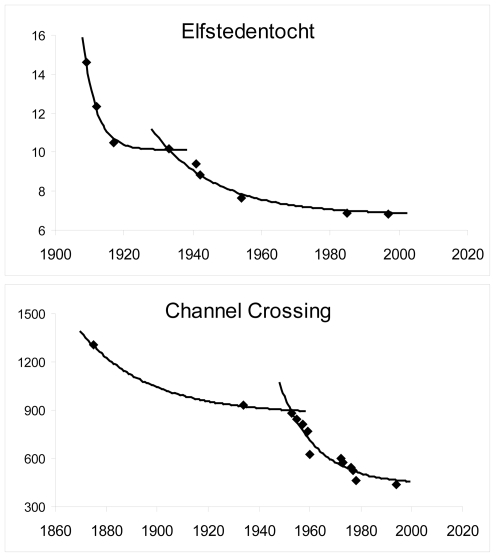
Model fitting for events with two progression periods. Performances of the Elfstedentocht speed skating race (normalized distance of 200 km) in hours and of the Channel crossing swimming in minutes.

**Figure 3:**
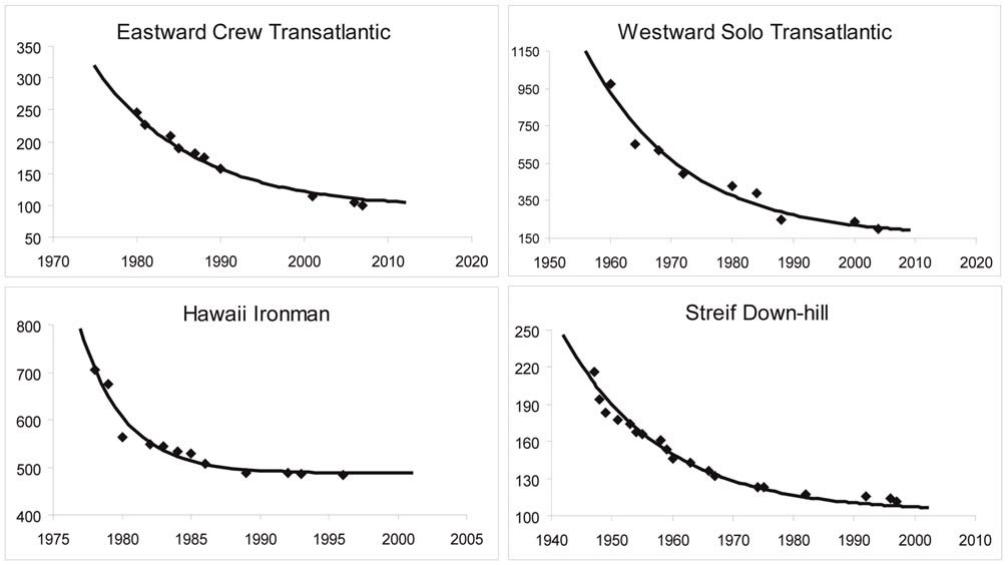
Model fitting for three events with a single progression period. Performances of the Eastward Crew and Westward Solo transatlantic records in hours, of the Hawaii Ironman in minutes and of the Streif down-hill in seconds.

**Figure 4:**
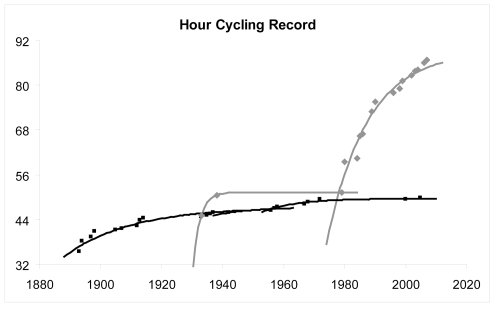
Model fitting for the hour cycling record. Hour cycling records in kilometres. Black lines and squares for record stated by the International Cyclist Union. Grey lines and diamonds for the record stated by the International Human Powered Vehicle Association.

The model fits progression periods, depending on the events' longevity, with high accuracy (*r^2^* = 0.95±0.07). A first progression period (XIX^th^ and beginning of XX^th^ century) was modeled for the older events (Oxford-Cambridge, Vasaloppet, speed ski record, ICU cycling: *r^2^* = 0.97±0.03, *a* = 1.93±0.96 and *β′* = 57.6±8.9%; **Figure 1**). A second progression period starts in the early XX^th^ century for seven events (the four previous ones plus Channel crossing, Elfstedentocht (**Figure 2**) and IHPVA cycling: *r^2^* ^= ^0.95±0.03, *a* = 0.97±0.45, *β′* = 73.15±16.8%). Modeled curves for the last period of all 10 events start in the middle of the XX^th^ century (*r^2^* ^= ^0.95±0.04, *a* = 1.28±0.51, *β′* = 94.5±7.2%**)**. The progression of the transatlantic records, the Hawaii Ironman and Streif down-hill are modeled by a mono-exponential curve (**Figure 3**). The curves for the hour cycling record progression according to the specific associations' rules (ICU and IHPVA) are compared in **Figure 4**.

The year when mean BP will be established at 99.95% of their asymptotic value is predicted to be 2049±32.1 years (**Table 2**).

**Table 2:** Coefficients and function fit for the asymptotic limit prediction.

Event	Date for asymptote achievement		*b*		*β*	*β′*
			
		*CI*		*CI*		
Eastward Crew transatlantic	2084	2050–2134	84.7	70.2–98.7	34.53	84.77
Westward Solo transatlantic	2107	2050–2206	155.8	66–246	23.89	77.71
Streif down-hill	2058	2045–2072	108.7	106–110	49.89	97.56
Speed ski record	2043	2021–2077	1.39	1.36–1.43	41.07	97.73
Hour cyling record IHPVA	2086	2057–2126	91.94	88.3–95.5	38.45	94.35
Oxford–Cambridge	2020	1990–2107	973.1	957–989	45.05	99.40
Vasaloppet	2022	1994–2096	12998	12650–13357	47.84	98.95
Elfstedentocht	2045	2027–2065	6.45	6.40–6.50	46.33	99.03
Ironmann Hawaï	2003	2000–2006	482.2	480–483	68.31	99.64
Channel crossing	2051	2017–2109	399.4	325–460	42.76	91.41
Hour cyling record ICU	2026	1984–2134	49.80	49.4–51.2	70.89	99.79

Year calculated for achieving 99.95% of the asymptote with confidence interval (CI); *b* is the asymptotic limit; *β* is the progression range from the beginning of the event expressed as a percentage of the predicted asymptotic limit; *β′* is the level of the actual BP expressed as a percentage of the asymptotic limit. Asymptotic limits *b* expressed in seconds for Oxford–Cambridge, Vasaloppet, Streif and speed ski record (for 100 meters); in minutes for the Hawaii Ironman and channel crossing; in hours for the Eastward, Westward transatlantic records and Elfstedentocht; in kilometres for the hour cycling records.

## Discussion

Our study is the first to model the performance evolution of outdoor sport events influenced by physiological, technological and environmental factors. Although the studied events largely differ in longevity, duration and physical environment (ice, snow, water, ground, air), the piecewise decaying exponential model describes a common evolutive pattern. Older events BPs follow a progression with high curvature coefficients suggesting the same rapid improvement in the early phase and a profile made up of two or three periods over the XIX^th^ and XX^th^ centuries. In addition, the initial *β* state that we estimated here is lower than the value previously calculated for Olympic sports (46% *vs* 60%), which may be due to the fact that here we took into account all records starting at day 0, whereas national Track & Field or Speed Skating competitions had already started long before the first Olympic games [1]. This emphasizes the particular status of these sport monuments in their own discipline.

Robert Fogel [18] previously used the term “techno-physiological evolution” to describe human health and anthropometric gains over the last three centuries. Similar improvements occured over the XX^th^ century (enhanced physical training, higher number of participants, nutritional practice, biological knowledge and medical advances) in sports like rowing [19], cross-country skiing [20], [21], speed skating [22] or cycling [23], which are sports with high aerobic requirements. Indeed, these events present similar progression patterns (*a* and *β* coefficients) than those obtained in Olympic disciplines performed in a controled environment and quantified by world records [1].

Predictions of the BP asymptotic limits from the piecewise exponential model suggest that the achievement of human limits may occur during the XXI^th^ century. Furthermore, our model suggests that the limits will be rapidly reached in events highly dependent on physiological capabilities, e.g. expected performance improvements over the next century in Oxford–Cambridge, Vasaloppet and Elfstedentocht are lower than 2%. The most recent of these events, the Hawaii Ironman, may have already achieved its asymptotic value (*β′* value: 99.64%) although it has been challenged since three decades only. The performance evolution for this event may be based on its rapidly growing popularity and benefits from the advances previously made in the three sports involved in triathlon (swimming, cycling, running). These results compared to those published for world records [1] suggest that environmental and climatic conditions, though influencing a year to year performance progression, do not modify the secular trend toward ultimate performance achievement.

Maximal boat speed in transatlantic records largely depends on environmental conditions (north Atlantic depression speed and numbers) rather than on individuals' physical power. Tools have been developed to control, at least partly, environmental factors for the athlete's benefits: e.g. navigation equipments allow for information to be transmitted to, or from, the boat in order to better take advantage of a major depression as well as of the lightest wind. Also, an increased knowledge and online computation now allows for a better use of sea streams. In addition, some events (speed ski, eastward transatlantic sailing, hour cycling) can be organized when optimal spatial and meteorological conditions are met in order to enhance probabilities to break a record. Choice for an event frequency and location may possibly influence BP occurrence and allow for a tight control of physical and climatic parameters. On the other side, the inability to organize the Elfstedentocht race due to the lack of thick ice between 1986 and 1997 and until now also demonstrates the major dependence of these competitions on climatic conditions [8]. Therefore, the progression of the Elfstedentocht performances could not benefit from the new skate technology that improved world skating records since 1998 [1], [24]. In a pre-determined place and date, Oxford defeated Cambridge in 2008 with the slowest time since 1947 and because of blustery and rough conditions the Cambridge boat sank in 1978 [5]. In 1987, the finishing time of the coldest Vasaloppet (−30°C) was 16 minutes longer than the record stated the year before. On another hand, due to extremely mild weather, the race was cancelled in 1990. However, as calculations integrate performances under-constraints, the particular environmental and climatic influences do not change the common pattern of performance evolution.

Our results demonstrate that higher progression rates remain plausible when rules allow for greater impact from technology. Sailing records present the lowest *β* and *β′* coefficients compared with rowing, cross-country skiing, triathlon or ICU cycling (group 2). The influence of technology is best demonstrated in the hour cycling record: the ICU “physiological” record limit is almost achieved at about 50 km/h, when using a vehicle similar to the Eddy Merckx bicycle but the predicted limit is much higher for the “technological” IHPVA value (91.94±3.58 km/h). In addition, the delay for achieving limits may be larger than for events highly influenced by physiological capabilities (2030 for group 2 vs. 2075 for group 1), as technology keeps modifying boats in transatlantic records (e.g. gauge, size and class, hull and keel types, sail surface), equipment for ski records (e.g. ski length and composition, aerodynamic helmets, latex or polyurethane suits) or aerodynamic surface in torpedo-like bikes [9], [10]. Although techno-physiological improvements allow for the enhancement of propulsive efficiency they remain under sport rules control: conception of rowing boats introduced carbon fiber use in 1972 and larger blades increased propulsive efficiency after 1991, but FISA banned sliding-rigger boats, which largely increased boat speed in order to limit technology influence and too expensive materials [4].

Anti-doping policies are elaborated to restrain pharmacological impacts within health perspectives [25] but technological advances are accepted within sport rules and identity (e.g. minimum weights for each class boat in rowing; Vasaloppet performed in classic style; Channel crossing with swim suits and hat without thermal protection or buoyancy capabilities) [4], [7], [15]. Conversely, the speed ski record, one of the fastest non motorized sport on land, seems to have reached its limits after 70 years of technological improvements under major environmental constraints (e.g. air penetration coefficient, snow quality, slope). Thus, if international federations, pushed by public demand and media coverage, want to develop sport on a pure performance basis with the fascination of newly established records, their need for technology will constantly increase. Swimming is the recent demonstration of such evolution with the introduction of swimming suits in 1998 and of second generation models in 2008 that allow for a 1 to 2% increase in speed [26]. However, close future and popularity of these sport monuments depend more on their history, identity and scenery than on continuous performance increase.

The present study suggests: *i)* the universality of the piecewise exponential model to describe the evolution of very short (1,4s) to very long sport performances (400 hours), six orders of magnitude apart; *ii)* a common law of progression in most sports toward ecophysiological limits; *iii)* major physiological constraints in the progression pattern of rowing, cross-country skiing, swimming, triathlon and ICU cycling record; *iv)* technology as a unique way to push back human physiological limits under environmental constraints, but with a major drawback: a constantly growing dependence on it.

In an accelerated process occurring over the XIX^th^ and XX^th^ centuries, sport performances took advantage of a common techno-physiological progression pathway [18] but soon reach their limits. Environnemental constraints now add to these development barriers. Human performance progression during the XXI^th^ century may essentially rely on technological improvements with its own reliance on energy and economy.
